# Supplementary material for: Urban density and depression during COVID-19 in Seoul: Moderating effects of social participation
Source: PLoS One. 2026 Jan 20;21(1):e0339040. doi: 10.1371/journal.pone.0339040 (PMC12818643; doi:10.1371/journal.pone.0339040)
Supplement: Attachment — Submitted filename: Response for reviewers comments(1106).docx [file pone.0339040.s002.docx]

**Required Changes for Acceptance**

**RC_1. Clarify Measurement of Depression**

**Reviewer's Comment:**

Multiple reviewers (R1, R2, R4) raised concerns regarding the single-item 10-point depression scale. Please provide clear information on the validation of this measure (line 290) and justify its use compared to standardized instruments (e.g., PHQ-9, CES-D).

**Response:**

Thank you for this important comment. We have now added validation evidence and justification for the single-item measure.

**1. Validation Evidence Added (Line 290)**

We added a paragraph citing empirical studies demonstrating validity of single-item depression measures in epidemiological research:

- **Zimmerman et al. (2006)**: Strong correlation with 18-item CUDOS (r=0.78, p<0.001) and clinician-rated CGI-S (r=0.64, p<0.001) among 562 outpatients
- **van Rijsbergen et al. (2014)**: 94% discriminative accuracy (AUC=0.94) against structured clinical interviews (SCID-I)
- **Mallon et al. (2002)**: 81% agreement with 14-item HADS in large epidemiological study

**2. Pandemic-Specific Justification Added (Line 295)**

We explained why this approach was appropriate for COVID-19 surveillance:

- **Rapid assessment**: Essential for timely public health response during evolving pandemic
- **Large-scale feasibility**: Minimized respondent burden (N=5,000 per wave)
- **Pandemic-specific focus**: Our measure explicitly assesses depressive symptoms "**due to the pandemic**," differentiating pandemic-attributed depression from general depression
- **International precedent**: Khajuria et al. (2021) successfully used single-item assessment among 2,527 healthcare workers across 41 countries during COVID-19

**3. Distinction from PHQ-9/CES-D**

Our measure serves different objectives than clinical instruments:

- **PHQ-9/CES-D**: Clinical diagnosis and comprehensive symptom assessment
- **Our measure**: Population-level surveillance of pandemic-attributed mental health impacts

This design aligns with municipal health monitoring objectives prioritizing rapid, large-scale assessment over detailed clinical evaluation.

**4. Limitations Acknowledged**

We revised the existing limitation statement to acknowledge measurement constraints while referencing the validation evidence:

**Original:** *"Third, the accuracy of measuring COVID-19-related depressive symptoms was limited. In this study, depressive symptoms were measured using a single-item 10-point scale..."*

**Revised:** *"Third, while our single-item 10-point depression scale has demonstrated validity in epidemiological research (as discussed in Methods section), it does not capture the multidimensional nature of depression assessed by comprehensive instruments such as the PHQ-9 or CES-D. Specifically, this measure may not fully reflect the complex dimensions of depressive symptoms caused by the COVID-19 pandemic. Future studies may benefit from incorporating more detailed depression assessments to examine specific symptom domains and enable clinical severity classification."*

Terminology and Clarity

RC_2. As Reviewer #2 suggests, replace vague phase terminology (“initial phase”) with precise year references (e.g., 2020 vs. 2021).

**Response:**

Thank you for this valuable suggestion to enhance clarity. We have systematically revised the entire manuscript to replace vague phase terminology with precise year references.

**1. Global Terminology Revision**

**All vague phase terminology has been replaced with year-specific references:**

- "initial phase" → "2020"
- "escalation phase" → "2021"
- "early pandemic" → "2020"
- "pandemic escalation" → "2021"

**2. Key Section Revisions**

**Materials and Methods (Lines 239-280):**

- Original: "...dividing it into two main phases: the initial and escalation phases"
- Revised: *"...dividing it into two periods: 2020 and 2021"*

**Results Section Headings:**

- "Results in the Initial Phase" → "Results in 2020"
- "Results in the Escalation Phase" → "Results in 2021"

**3. Updated Table Titles**

- Table 4: "Results of COVID-19-Related Depressive Symptoms in 2020"
- Table 5: "Results of COVID-19-Related Depressive Symptoms in 2021"

**4. Contextual Phase References**

Where analytical context requires phase references, we use parenthetical notation:

- Example: "During 2020 (initial phase of the pandemic)"
- This maintains clarity while preserving the analytical framework

RC_3. Clarify whether “cumulative odds” refers specifically to proportional odds in ordinal logistic regression (R2).

**Response:**

Thank you for requesting clarification on the terminology. Yes, we are referring to cumulative odds in the context of the proportional odds model.

In the ordered logistic regression analysis used in this study:

- We calculate cumulative odds, defined as P(Y ≤ j) / P(Y > j), which represents the ratio of the probability of being at or below a particular category versus being above that category for each threshold j

- The model operates under the proportional odds assumption, meaning that the effect coefficients (β) remain constant across all cumulative logits, while only the threshold parameters (intercepts) vary across categories

We have revised the manuscript to clarify this terminology. Specifically, we have:

1. Explicitly noted that the ordered logistic model is a proportional odds model

2. Added a precise definition of cumulative odds with the mathematical expression P(Y ≤ j) / P(Y > j)

3. Explained the proportional odds assumption underlying the model

These revisions ensure that readers understand both the specific type of odds being modeled (cumulative odds) and the key assumption (proportional odds) that characterizes this analytical approach.

Methods Transparency

RC_4. Expand details on how “mobility density” was calculated (lines 318–326, R1).

**Response:**

Thank you for this important request for methodological transparency. We have expanded the description of urban density measures, including population mobility, in the Methods section.

**Mobility Density Measurement**

Mobility density was calculated using Seoul's Living Population dataset, which provides daily aggregated mobile population counts at the district level. This dataset is publicly available through the Seoul Metropolitan Government's open data platform. The measure represents the daily maximum mobile population in each district divided by the district's geographic area (km²), expressed as persons per square kilometer.

**Data Source and Collection Methodology**

The Seoul Living Population dataset is derived from KT (Korea Telecom) LTE network data, which captures real-time location patterns through mobile base station signals. The system tracks population movements on an hourly basis, distinguishing between nighttime residential locations and daytime activity locations (e.g., workplaces, commercial areas).

**Residence Classification Algorithm**

An individual's residence is algorithmically determined using the following criteria:

- Continuous presence in a location for at least 4 hours between midnight and 6:00 AM
- Consistent repetition of this pattern over a minimum 14-day observation period
- Coverage includes Korean nationals and long-term foreign residents with active mobile subscriptions

**Rationale for Using Daily Maximum Values**

Rather than daily averages, we used the daily maximum mobile population—the highest hourly count recorded each day. This approach offers three key advantages:

1. **Peak exposure assessment**: Captures moments of highest infection transmission risk during rush hours, lunch periods, and other times of concentrated human contact
2. **Infrastructure stress points**: Reflects maximum congestion levels in public transportation and shared spaces where disease transmission is most likely
3. **Epidemiological validity**: Prior research demonstrates that peak mobility volumes are stronger predictors of infectious disease transmission than average daily movements

**Calculation:**

Mobility Density = Daily Maximum Mobile Population (persons) / District Area (km²)

RC_5. Provide information on sample size, demographic characteristics, and the source of the data (R3).

**Response:**

We have enhanced the manuscript with detailed information about sample size, demographics, and data sources.

**1. Sample Size Clarification**

- **Original:** " The Seoul Citizen Survey targeted 5,000 individuals aged 15 years and older residing in Seoul..."
- ***Revised:*** *"* *The Seoul Citizen Survey annually targeted 5,000 individuals aged 15 years and older residing in Seoul, yielding a total sample of 10,000 participants across the two study periods (2020: n=5,000; 2021: n=5,000)."*

**2. Demographic Characteristics**

- **Original:** "These characteristics included sex (male, female), age, educational level..."
- ***Revised:*** *"* *Participants were categorized into three age groups for analysis: young adults (19-34 years), middle-aged adults (35-64 years), and older adults (65 years and above), with sample distributions detailed in Table 2. These characteristics included sex (male, female), age, educational level..."*

**3. Table 2 Enhancement**

- **Original:** "Table 2 illustrates the differences in COVID-19-related depressive symptoms according to year and age group."
- ***Revised:*** *Table 2 presents the sample distribution and differences in COVID-19-related depressive symptoms by pandemic phase and age group. In 2020, the sample comprised 1,227 young adults (19-34 years), 2,695 middle-aged adults (35-64 years), and 1,080 older adults (≥65 years). In 2021, the distribution was 1,302 young adults, 2,697 middle-aged adults, and 1,001 older adults.*

RC_6. Discussion of Limitations

Strengthen the discussion of limitations (R1, R4):

• Cross-sectional design and lack of longitudinal data.

• Generalizability limited to Seoul’s unique urban context.

• Simplified, unidimensional measurement of depression.

These should be explicitly acknowledged in the Discussion (lines 578–590).

**Response:**

Thank you for this critical feedback. We have substantially strengthened our limitations discussion to explicitly acknowledge these methodological constraints with the following key enhancements:

1. Cross-sectional Design Limitation

Clarified that our two-timepoint data represent independent samples rather than repeated measures, explicitly stating this limits causal inference and that our findings demonstrate associations, not causality. Added specific recommendation for longitudinal studies to establish causal relationships.

2. Limited Generalizability

Added specific contextual details characterizing Seoul's unique urban environment (population density of 16,000 persons/km²; extensive public transportation system) to help readers understand why generalization to other contexts requires caution. Recommended future research across diverse urban settings.

3. Single-item Depression Measure Limitation

Reframed from vague "accuracy limitation" to explicitly acknowledge the measure does not capture depression's multidimensional nature. Distinguished our epidemiological surveillance objective from clinical assessment, while recommending comprehensive instruments (PHQ-9, CES-D) for future research to examine specific symptom domains and severity classification.

These revisions transform our original brief limitation statements into a comprehensive discussion that transparently acknowledges methodological constraints while providing concrete directions for future research.

RC_7. Interpretation of Results

Avoid causal language (R4). Reframe results to emphasize associations rather than causality, given the cross-sectional design.

**Response:**

Thank you for this crucial methodological point. We have systematically revised the entire manuscript to remove causal language and emphasize associations consistent with our cross-sectional design.

**1. Abstract Revisions (Lines 33-40)**

First finding:

- **Original:** "...were consistently associated with increased depression throughout the pandemic"
- **Revised:** *"...were consistently associated with higher levels of depression across both study periods (2020 and 2021)"*

Second finding:

- **Original:** " social participation demonstrated a buffering effect, mitigating the depressive impact of urban density characteristics regardless of the pandemic phase."
- **Revised:** *" Second, social participation showed a significant moderating association with the relationship between urban density characteristics and depression, regardless of the pandemic "*

**2. Results and Discussion Revisions**

Temporal patterns:

- **Original:** " This finding suggests the possibility of adapting to the pandemic over time. "
- **Revised:** *"* *This finding indicates a temporal decline in COVID-19-related depressive symptoms during the study period. "*

Policy associations:

- **Original:** " ..., such as encouraging people to stay at home, likely led to increased residential density "
- **Revised:** " *..., such as encouraging people to stay at home,* *were associated with increased residential density "*
- **Original:** " coupled with stay-at-home policies, can amplify pandemic-related depressive symptoms. "
- **Revised:** *" coupled with stay-at-home policies,* *were associated with higher pandemic-related depressive symptoms "*
- **Original:** "These results suggest that social participation mitigates infection-related anxiety arising from urban density."
- **Revised:** *"These results suggest that social participation* *was associated with lower infection-related anxiety arising from urban density."*
- **Original:** "Active social interactions appear to alleviate depressive symptoms even in high-density environments."
- **Revised:** *"Active social interactions* *were associated with lower depressive symptoms even in high-density environments."*

**4. Conclusion Revisions**

- **Original:** "This suggests that dense urban environments can amplify anxiety about infections."
- **Revised:** *"This suggests that dense urban environments* *were associated with higher anxiety about infections."*
- **Original:** "playing a role in reducing the increase in depressive symptoms caused by high urban density"
- **Revised:** *"playing a role in reducing the increase in depressive symptoms associated with high urban density"*

Recommended Revisions

RR_1. Title

Reviewer #1 suggests a more concise title, while Reviewer #3 finds the current title clear and appropriate. A balanced option would be to retain the essential elements but simplify phrasing, e.g., “Urban Density, Social Participation, and Depression during COVID-19 in Seoul”.

**Response:**

Thank you for the valuable feedback regarding the manuscript title. We have carefully considered both reviewers' comments and your recommendation for a compromise approach. We have revised the title to: "Urban density and depression during COVID-19 in Seoul: Moderating effects of social participation"

This revision maintains all key elements (urban density, depression, COVID-19, Seoul, and social participation) while achieving greater conciseness by removing detailed methodological aspects (age and phase differences). These important distinctions remain thoroughly addressed in the abstract and main text.

We believe this balanced approach satisfies both the call for brevity and the need for clarity in communicating our study's core contribution.

Abstract and Introduction

RR_2. Briefly expand the methods description in the Abstract (R1).

**Response:**

We appreciate this valuable suggestion. We have expanded the methods description in the Abstract to provide greater clarity and transparency about our study design and analytical approach. The revised Abstract now includes:

1) The measurement method for depressive symptoms (10-point self-report scale)

2) Specific urban density variables examined (population mobility density, residential density, and public transportation congestion)

3) Data sources for these variables (mobile phone signal data and building registries)

4) The analytical strategy of using separate models for three age groups

These additions provide readers with a clearer understanding of our methodological approach without making the Abstract overly technical or exceeding the 300-word limit required by PLOS ONE.

RR_3. More clearly specify the literature gaps addressed by this study in the Introduction (R1).

**Response:**

We sincerely thank the reviewer for this critical suggestion. We acknowledge that while our original manuscript did identify research gaps in the Literature Review section, these were not sufficiently prominent or specific enough for readers to clearly recognize them. The gaps were embedded within the literature discussion rather than being explicitly articulated as distinct knowledge deficits that our study addresses.

To address this concern, we have substantially revised the final portion of the Literature Review section (which serves as the transition to our study's objectives) to make the research gaps far more explicit and concrete. Specifically, we have:

1) Restructured the gap discussion from a single, general statement into a dedicated paragraph that clearly enumerates three specific gaps with supporting citations.

2) For each gap, we now explicitly contrast what existing studies have examined versus what remains unknown, using concrete references:

• Temporal gap: Although some studies tracked general mental health changes [29-32], most urban density studies used single-timepoint cross-sectional designs [36-42], precluding analysis of how urban density-depression relationships evolved across different pandemic phases. Our study addresses this by comparing the initial phase (2020) and escalation phase (2021).

• Age-specific gap: While age differences in COVID-19 vulnerability are well-documented [21-27, 33], existing urban density studies have not systematically examined whether urban density effects on depression vary across age groups [36-42]. Our study addresses this through separate analyses for young, middle-aged, and older adults.

• Moderating mechanism gap: Although social participation protects mental health during crises [47-49], its potential to buffer urban density stressors during infectious disease outbreaks remains unexplored. Our study addresses this through interaction term analyses.

3) Enhanced the logical flow by reorganizing the final section into three distinct paragraphs: (a) research aim and existing literature summary, (b) explicit specification of three research gaps with citations demonstrating what has/has not been studied, and (c) our study's approach and research questions.

4) Added a transitional statement explaining why addressing these gaps is essential for developing age-specific and phase-appropriate urban mental health interventions during pandemics.

The revised Literature Review section now makes it immediately clear to readers what specific knowledge gaps exist in the literature and precisely how our study fills these gaps. The gaps are no longer implicit but are stated explicitly with concrete evidence from prior studies.

Discussion and Conclusion

RR_4. Add practical recommendations for policymakers based on your findings (R1, R3).

**Response:**

We sincerely thank both reviewers and the editor for this valuable suggestion to strengthen the practical applicability of our findings. We have substantially expanded the Conclusion section by adding three comprehensive paragraphs that translate our empirical results into actionable policy recommendations.

The additions address three key domains:

First, we now provide specific recommendations for urban planning and environmental interventions to mitigate density-related mental health risks during pandemics. These include enhancing access to diverse green spaces (plazas, parks, riverside greenways, mountain belts characteristic of Seoul's landscape), implementing public transportation measures to reduce crowding (increased frequency, real-time occupancy monitoring, enhanced sanitation protocols), and adopting "15-minute city" concepts that strategically locate essential infrastructure within residential neighborhoods to minimize population mobility.

Second, we outline phase-adaptive mental health policies that evolve with pandemic progression. We specify that early-phase interventions should focus on reducing acute fears through clear risk communication and immediate psychological support, while later-phase policies should emphasize rebuilding social networks and facilitating community reintegration programs. Throughout all phases, we recommend actively facilitating safe social engagement opportunities through outdoor activities, neighborhood networks, and digital connectivity programs.

Third, we provide age-specific recommendations addressing the heightened vulnerability of older adults demonstrated in our study. These include establishing accessible community centers within walking distance, implementing volunteer visitor programs, creating safe outdoor spaces designed for older adult mobility, and conducting proactive outreach to identify at-risk older adults in high-density neighborhoods [26,54-57].

These policy recommendations are directly grounded in our study's key findings—particularly the moderating role of social participation, the age-specific vulnerability patterns, and the phase-dependent changes in depression—and are supported by relevant literature. We believe these additions substantially enhance the manuscript's relevance for evidence-based policymaking during infectious disease outbreaks.

RR_5. Consider including multidimensional depression measures (PHQ-9, CES-D) as a recommendation for future research (R1, R4).

**Response:**

We appreciate this constructive suggestion from both reviewers. We have incorporated this recommendation into the Limitations section of the Discussion.

The revised text now:

1) Acknowledges that while our single-item 10-point depression scale has demonstrated validity in epidemiological research (as justified in the Methods section), it does not capture the multidimensional nature of depression assessed by comprehensive instruments.

2) Explicitly mentions PHQ-9 and CES-D as examples of multidimensional measures that could provide more nuanced assessment.

3) Explains the specific advantages of using such instruments: they can examine specific symptom domains (e.g., cognitive, somatic, affective symptoms) and enable clinical severity classification, which our single-item measure cannot do.

4) Provides a clear recommendation for future research: "Future studies may benefit from incorporating more detailed depression assessments to examine specific symptom domains and enable clinical severity classification." This addition strengthens our manuscript by acknowledging the measurement limitation while providing actionable guidance for future researchers in this area.

Tables and Redundancy

RR_6. Consider moving Table 1 to the supplementary materials (R1).

**Response:**

We appreciate the reviewer's suggestion to streamline the manuscript. However, we respectfully prefer to retain Table 1 in the main text for the following reasons:

1) Table 1 is fundamental to understanding our study design. The classification of pandemic phases (Initial phase: 1st+2nd waves vs. Escalation phase: 3rd+4th waves) shown in this table is essential for interpreting all subsequent analyses, particularly the comparison between Table 4 (2020 results) and Table 5 (2021 results).

2) The table serves a different function than the accompanying text. While the text provides narrative context and interpretation, Table 1 enables readers to quickly compare key epidemiological metrics (confirmed cases, deaths, fatality rates) across five waves at a glance. This visual comparison is difficult to achieve through text alone.

3) Table 1 is closely integrated with Figure 2, as indicated by the joint citation "(Table 1; Fig 2)". Moving the table to supplementary materials would disrupt this important visual-tabular connection that helps readers understand the pandemic progression in Seoul.

For these reasons, we believe Table 1 provides essential context that warrants its inclusion in the main text.

RR_7. Reduce repetition in the background section (lines 127–151 and 183–204, R1).

**Response:**

We sincerely thank the reviewer for this helpful observation. Upon careful review, we identified two areas in the Literature Review section where content was repetitive and could be more concisely presented.

We have revised these sections as follows:

1) Age-specific effects section (lines 127-151): The original manuscript contained three separate paragraphs discussing the mental health impacts on different age groups during COVID-19. The first paragraph introduced age differences, the second focused on middle-aged adults (particularly parents), the third on older adults, followed by a summary sentence. While each presented valuable information, they contained overlapping themes regarding vulnerability and social isolation.

We have consolidated these into a single, cohesive paragraph that presents the age-specific findings more efficiently. The revised paragraph maintains all key findings and citations [21-27] while eliminating redundant introductory and transitional phrases. This consolidation improves readability while preserving the substantive contrast between middle-aged adults (who experienced complex parenting-related stressors) and older adults (who faced greater health risks and social isolation).

2) Urban density studies section (lines 183-204): The original text sequentially listed findings from individual studies across multiple countries (United States, Belgium, Netherlands), with each sentence or pair of sentences presenting similar conclusions about heightened mental health risks in urban areas. This resulted in repetitive phrasing such as "A study found... Additionally... Moreover... A study conducted... Similarly..."

We have synthesized these findings into a single, unified paragraph that presents the collective evidence more efficiently. The revised text groups U.S. studies together [36-38] and European studies together [39-42], clearly demonstrating the consistent pattern across multiple countries while maintaining all original citations. This approach eliminates redundancy while strengthening the overall argument by emphasizing the consistency of findings across diverse contexts.

These revisions reduce approximately 180 words from the Literature Review section while preserving all substantive content, improving both conciseness and readability. The logical flow and argumentation remain intact, with enhanced clarity.

References

RR_8. Reviewer #1 recommends including da Cruz et al. (2022) to strengthen the discussion of social interactions and physical activity in older adults. This is appropriate and encouraged.

**Response:**

We thank the reviewer for this valuable suggestion. We have incorporated this reference as [56] in the age-specific policy recommendations section of the Conclusion (third paragraph).

Specifically, we have added the following sentence after discussing priority interventions for older populations in dense urban areas: "When community activities are restricted due to infection control measures, supporting home-based physical activity programs can provide alternative mental health benefits for isolated older adults [56]."

This addition enhances the practical applicability of our policy recommendations by acknowledging that during periods of strict infection control—such as lockdowns or high transmission phases—policymakers need alternative strategies to support older adults' mental health when community-based social participation is temporarily infeasible.

This incorporation strengthens our policy recommendations by addressing both optimal scenarios (community engagement) and constrained scenarios (home-based alternatives), thereby providing more comprehensive guidance for policymakers navigating the complexities of mental health protection during infectious disease outbreaks.

Reviewer #1

Reviewer #1: Thank you to the authors and the editor for the opportunity to review this study titled Urban density and depression during COVID-19 in Seoul: Age and phase differences with the moderating role of social participation. This work provides valuable empirical analysis on the effect of urban density and social participation on depressive symptoms during the COVID-19 pandemic in Seoul, addressing differences across phases and age groups.

Strengths
Relevance of the topic (lines 17–35): The article explores a highly relevant issue, delving into the link between urban density and mental health during the pandemic. This contributes significantly to the understanding of the complex interactions between urban environments and psychological well-being.

Robust methodology (lines 237–389): The use of ordered logistic regression models and the inclusion of variables such as residential density, mobility, and transportation congestion strengthen the validity of the results.

Impact of social interactions (lines 495–554): The article emphasizes the role of social participation as a moderator, highlighting effects specific to different age groups, which makes a significant contribution to the existing literature.

Suggested Modifications

1. Title (lines 1–6): Consider a shorter, more concise title, such as "Effects of Urban Density and Social Participation on COVID-19-Related Depression in Seoul."

**Response:**

We appreciate the reviewer's suggestion for a more concise title. In response to this feedback and the editor's recommendation, we have revised the title from:

"Urban density and depression during COVID-19 in Seoul: Age and phase differences with the moderating role of social participation"

to:

"Urban density and depression during COVID-19 in Seoul: Moderating effects of social participation"

This revision achieves greater conciseness by removing the detailed methodological descriptor ("Age and phase differences with") while retaining all essential research elements: urban density, depression, COVID-19, Seoul, and social participation. We believe this balanced approach addresses the reviewer's concern for brevity while maintaining clarity about the study's core focus on the moderating role of social participation.

2. Abstract (lines 17–38): Briefly expand the reference to the methodology for greater clarity.

**Response:**

We appreciate this valuable suggestion. We have expanded the methods description in the Abstract to provide greater clarity and transparency about our study design and analytical approach. The revised Abstract now includes:

1) The measurement method for depressive symptoms (10-point self-report scale)

2) Specific urban density variables examined (population mobility density, residential density, and public transportation congestion)

3) Data sources for these variables (mobile phone signal data and building registries)

4) The analytical strategy of using separate models for three age groups

These additions provide readers with a clearer understanding of our methodological approach without making the Abstract overly technical or exceeding the 300-word limit required by PLOS ONE.

3. Introduction (lines 39–109): Clearly specify gaps in the current literature and how this study addresses them.

**Response:**

We sincerely thank the reviewer for this critical suggestion. We acknowledge that while our original manuscript did identify research gaps in the Literature Review section, these were not sufficiently prominent or specific enough for readers to clearly recognize them. The gaps were embedded within the literature discussion rather than being explicitly articulated as distinct knowledge deficits that our study addresses.

To address this concern, we have substantially revised the final portion of the Literature Review section (which serves as the transition to our study's objectives) to make the research gaps far more explicit and concrete. Specifically, we have:

1) Restructured the gap discussion from a single, general statement into a dedicated paragraph that clearly enumerates three specific gaps with supporting citations.

2) For each gap, we now explicitly contrast what existing studies have examined versus what remains unknown, using concrete references:

• Temporal gap: Although some studies tracked general mental health changes [29-32], most urban density studies used single-timepoint cross-sectional designs [36-42], precluding analysis of how urban density-depression relationships evolved across different pandemic phases. Our study addresses this by comparing the initial phase (2020) and escalation phase (2021).

• Age-specific gap: While age differences in COVID-19 vulnerability are well-documented [21-27, 33], existing urban density studies have not systematically examined whether urban density effects on depression vary across age groups [36-42]. Our study addresses this through separate analyses for young, middle-aged, and older adults.

• Moderating mechanism gap: Although social participation protects mental health during crises [47-49], its potential to buffer urban density stressors during infectious disease outbreaks remains unexplored. Our study addresses this through interaction term analyses.

3) Enhanced the logical flow by reorganizing the final section into three distinct paragraphs: (a) research aim and existing literature summary, (b) explicit specification of three research gaps with citations demonstrating what has/has not been studied, and (c) our study's approach and research questions.

4) Added a transitional statement explaining why addressing these gaps is essential for developing age-specific and phase-appropriate urban mental health interventions during pandemics.

The revised Literature Review section now makes it immediately clear to readers what specific knowledge gaps exist in the literature and precisely how our study fills these gaps. The gaps are no longer implicit but are stated explicitly with concrete evidence from prior studies.

Materials and Methods Section (lines 237–389):

4. Line 290: Add information on the validation of the depression measurement scale.

**Response:**

Thank you for this important comment. We have now added validation evidence and justification for the single-item measure.

**1. Validation Evidence Added (Line 290)**

We added a paragraph citing empirical studies demonstrating validity of single-item depression measures in epidemiological research:

- **Zimmerman et al. (2006)**: Strong correlation with 18-item CUDOS (r=0.78, p<0.001) and clinician-rated CGI-S (r=0.64, p<0.001) among 562 outpatients
- **van Rijsbergen et al. (2014)**: 94% discriminative accuracy (AUC=0.94) against structured clinical interviews (SCID-I)
- **Mallon et al. (2002)**: 81% agreement with 14-item HADS in large epidemiological study

**2. Pandemic-Specific Justification Added (Line 295)**

We explained why this approach was appropriate for COVID-19 surveillance:

- **Rapid assessment**: Essential for timely public health response during evolving pandemic
- **Large-scale feasibility**: Minimized respondent burden (N=5,000 per wave)
- **Pandemic-specific focus**: Our measure explicitly assesses depressive symptoms "**due to the pandemic**," differentiating pandemic-attributed depression from general depression
- **International precedent**: Khajuria et al. (2021) successfully used single-item assessment among 2,527 healthcare workers across 41 countries during COVID-19

**3. Distinction from PHQ-9/CES-D**

Our measure serves different objectives than clinical instruments:

- **PHQ-9/CES-D**: Clinical diagnosis and comprehensive symptom assessment
- **Our measure**: Population-level surveillance of pandemic-attributed mental health impacts

This design aligns with municipal health monitoring objectives prioritizing rapid, large-scale assessment over detailed clinical evaluation.

**4. Limitations Acknowledged**

We revised the existing limitation statement to acknowledge measurement constraints while referencing the validation evidence:

**Original:** *"Third, the accuracy of measuring COVID-19-related depressive symptoms was limited. In this study, depressive symptoms were measured using a single-item 10-point scale..."*

**Revised:** *"Third, while our single-item 10-point depression scale has demonstrated validity in epidemiological research (as discussed in Methods section), it does not capture the multidimensional nature of depression assessed by comprehensive instruments such as the PHQ-9 or CES-D. Specifically, this measure may not fully reflect the complex dimensions of depressive symptoms caused by the COVID-19 pandemic. Future studies may benefit from incorporating more detailed depression assessments to examine specific symptom domains and enable clinical severity classification."*

5. Lines 318–326: Provide more details about the calculation of mobility density to improve transparency.

**Response:**

Thank you for this important request for methodological transparency. We have expanded the description of urban density measures, including population mobility, in the Methods section.

**Mobility Density Measurement**

Mobility density was calculated using Seoul's Living Population dataset, which provides daily aggregated mobile population counts at the district level. This dataset is publicly available through the Seoul Metropolitan Government's open data platform. The measure represents the daily maximum mobile population in each district divided by the district's geographic area (km²), expressed as persons per square kilometer.

**Data Source and Collection Methodology**

The Seoul Living Population dataset is derived from KT (Korea Telecom) LTE network data, which captures real-time location patterns through mobile base station signals. The system tracks population movements on an hourly basis, distinguishing between nighttime residential locations and daytime activity locations (e.g., workplaces, commercial areas).

**Residence Classification Algorithm**

An individual's residence is algorithmically determined using the following criteria:

- Continuous presence in a location for at least 4 hours between midnight and 6:00 AM
- Consistent repetition of this pattern over a minimum 14-day observation period
- Coverage includes Korean nationals and long-term foreign residents with active mobile subscriptions

**Rationale for Using Daily Maximum Values**

Rather than daily averages, we used the daily maximum mobile population—the highest hourly count recorded each day. This approach offers three key advantages:

4. **Peak exposure assessment**: Captures moments of highest infection transmission risk during rush hours, lunch periods, and other times of concentrated human contact
5. **Infrastructure stress points**: Reflects maximum congestion levels in public transportation and shared spaces where disease transmission is most likely
6. **Epidemiological validity**: Prior research demonstrates that peak mobility volumes are stronger predictors of infectious disease transmission than average daily movements

**Calculation:**

Mobility Density = Daily Maximum Mobile Population (persons) / District Area (km²)

Discussion (lines 558–596):

6. Line 578: Strengthen the analysis of limitations by emphasizing how the results may differ in rural contexts or cities with lower densities.

**Response:**

This concern is addressed in our response to Comment 12 below, where we have substantially expanded the generalizability limitation to explicitly discuss how our findings from Seoul—a high-density metropolitan city—may differ in rural contexts or cities with lower densities.

7. Line 590: Suggest approaches to integrate multidimensional scales for depression in future studies.
**Response:**

We appreciate this constructive suggestion from both reviewers. We have incorporated this recommendation into the Limitations section of the Discussion.

The revised text now:

1) Acknowledges that while our single-item 10-point depression scale has demonstrated validity in epidemiological research (as justified in the Methods section), it does not capture the multidimensional nature of depression assessed by comprehensive instruments.

2) Explicitly mentions PHQ-9 and CES-D as examples of multidimensional measures that could provide more nuanced assessment.

3) Explains the specific advantages of using such instruments: they can examine specific symptom domains (e.g., cognitive, somatic, affective symptoms) and enable clinical severity classification, which our single-item measure cannot do.

4) Provides a clear recommendation for future research: "Future studies may benefit from incorporating more detailed depression assessments to examine specific symptom domains and enable clinical severity classification." This addition strengthens our manuscript by acknowledging the measurement limitation while providing actionable guidance for future researchers in this area.

8. Conclusion (lines 558–596): Include practical recommendations for policymakers.

**Response:**

We sincerely thank both reviewers and the editor for this valuable suggestion to strengthen the practical applicability of our findings. We have substantially expanded the Conclusion section by adding three comprehensive paragraphs that translate our empirical results into actionable policy recommendations.

The additions address three key domains:

First, we now provide specific recommendations for urban planning and environmental interventions to mitigate density-related mental health risks during pandemics. These include enhancing access to diverse green spaces (plazas, parks, riverside greenways, mountain belts characteristic of Seoul's landscape), implementing public transportation measures to reduce crowding (increased frequency, real-time occupancy monitoring, enhanced sanitation protocols), and adopting "15-minute city" concepts that strategically locate essential infrastructure within residential neighborhoods to minimize population mobility.

Second, we outline phase-adaptive mental health policies that evolve with pandemic progression. We specify that early-phase interventions should focus on reducing acute fears through clear risk communication and immediate psychological support, while later-phase policies should emphasize rebuilding social networks and facilitating community reintegration programs. Throughout all phases, we recommend actively facilitating safe social engagement opportunities through outdoor activities, neighborhood networks, and digital connectivity programs.

Third, we provide age-specific recommendations addressing the heightened vulnerability of older adults demonstrated in our study. These include establishing accessible community centers within walking distance, implementing volunteer visitor programs, creating safe outdoor spaces designed for older adult mobility, and conducting proactive outreach to identify at-risk older adults in high-density neighborhoods [26,54-57].

These policy recommendations are directly grounded in our study's key findings—particularly the moderating role of social participation, the age-specific vulnerability patterns, and the phase-dependent changes in depression—and are supported by relevant literature. We believe these additions substantially enhance the manuscript's relevance for evidence-based policymaking during infectious disease outbreaks.

9. Table 1 (lines 278–279): Consider moving this table to the supplementary materials, as much of the information is already described in the text.

**Response:**

We appreciate the reviewer's suggestion to streamline the manuscript. However, we respectfully prefer to retain Table 1 in the main text for the following reasons:

1) Table 1 is fundamental to understanding our study design. The classification of pandemic phases (Initial phase: 1st+2nd waves vs. Escalation phase: 3rd+4th waves) shown in this table is essential for interpreting all subsequent analyses, particularly the comparison between Table 4 (2020 results) and Table 5 (2021 results).

2) The table serves a different function than the accompanying text. While the text provides narrative context and interpretation, Table 1 enables readers to quickly compare key epidemiological metrics (confirmed cases, deaths, fatality rates) across five waves at a glance. This visual comparison is difficult to achieve through text alone.

3) Table 1 is closely integrated with Figure 2, as indicated by the joint citation "(Table 1; Fig 2)". Moving the table to supplementary materials would disrupt this important visual-tabular connection that helps readers understand the pandemic progression in Seoul.

For these reasons, we believe Table 1 provides essential context that warrants its inclusion in the main text.

10. Repetitions (lines 127–151 and 183–204): Some paragraphs in the background section could be summarized to avoid redundancy.

**Response:**

We sincerely thank the reviewer for this helpful observation. Upon careful review, we identified two areas in the Literature Review section where content was repetitive and could be more concisely presented.

We have revised these sections as follows:

1) Age-specific effects section (lines 127-151): The original manuscript contained three separate paragraphs discussing the mental health impacts on different age groups during COVID-19. The first paragraph introduced age differences, the second focused on middle-aged adults (particularly parents), the third on older adults, followed by a summary sentence. While each presented valuable information, they contained overlapping themes regarding vulnerability and social isolation.

We have consolidated these into a single, cohesive paragraph that presents the age-specific findings more efficiently. The revised paragraph maintains all key findings and citations [21-27] while eliminating redundant introductory and transitional phrases. This consolidation improves readability while preserving the substantive contrast between middle-aged adults (who experienced complex parenting-related stressors) and older adults (who faced greater health risks and social isolation).

2) Urban density studies section (lines 183-204): The original text sequentially listed findings from individual studies across multiple countries (United States, Belgium, Netherlands), with each sentence or pair of sentences presenting similar conclusions about heightened mental health risks in urban areas. This resulted in repetitive phrasing such as "A study found... Additionally... Moreover... A study conducted... Similarly..."

We have synthesized these findings into a single, unified paragraph that presents the collective evidence more efficiently. The revised text groups U.S. studies together [36-38] and European studies together [39-42], clearly demonstrating the consistent pattern across multiple countries while maintaining all original citations. This approach eliminates redundancy while strengthening the overall argument by emphasizing the consistency of findings across diverse contexts.

These revisions reduce approximately 180 words from the Literature Review section while preserving all substantive content, improving both conciseness and readability. The logical flow and argumentation remain intact, with enhanced clarity.

11. Encourage the authors to include the following reference, which is particularly relevant for the role of physical activity in older adults’ mental health during the pandemic. It could be added in the Discussion section (line 560), supporting the importance of social interactions:

da Cruz, W. M., D'Oliveira, A., Dominski, F. H., Diotaiuti, P., & Andrade, A. (2022). Mental health of older people in social isolation: the role of physical activity at home during the COVID-19 pandemic. Sport Sciences for Health, 18(2), 597–602. <https://doi.org/10.1007/s11332-021-00825-9>

**Response:**

We thank the reviewer for this valuable suggestion. We have incorporated this reference as [56] in the age-specific policy recommendations section of the Conclusion (third paragraph).

Specifically, we have added the following sentence after discussing priority interventions for older populations in dense urban areas: "When community activities are restricted due to infection control measures, supporting home-based physical activity programs can provide alternative mental health benefits for isolated older adults [56]."

This addition enhances the practical applicability of our policy recommendations by acknowledging that during periods of strict infection control—such as lockdowns or high transmission phases—policymakers need alternative strategies to support older adults' mental health when community-based social participation is temporarily infeasible.

This incorporation strengthens our policy recommendations by addressing both optimal scenarios (community engagement) and constrained scenarios (home-based alternatives), thereby providing more comprehensive guidance for policymakers navigating the complexities of mental health protection during infectious disease outbreaks.

12. The authors have not sufficiently addressed the following limitations:

- Cross-sectional nature of the data (line 578): A longitudinal design could provide greater insights into temporal changes.

- Geographical contextualization (line 583): The findings may not be generalizable to contexts outside Seoul.

- Unidimensional measurement of depression (line 590): Incorporating validated scales such as the PHQ-9 would allow for a more comprehensive assessment.

**Response:**

Thank you for this critical feedback. We have substantially strengthened our limitations discussion to explicitly acknowledge these methodological constraints with the following key enhancements:

1. Cross-sectional Design Limitation

Clarified that our two-timepoint data represent independent samples rather than repeated measures, explicitly stating this limits causal inference and that our findings demonstrate associations, not causality. Added specific recommendation for longitudinal studies to establish causal relationships.

2. Limited Generalizability

Added specific contextual details characterizing Seoul's unique urban environment (population density of 16,000 persons/km²; extensive public transportation system) to help readers understand why generalization to other contexts requires caution. Recommended future research across diverse urban settings.

3. Single-item Depression Measure Limitation

Reframed from vague "accuracy limitation" to explicitly acknowledge the measure does not capture depression's multidimensional nature. Distinguished our epidemiological surveillance objective from clinical assessment, while recommending comprehensive instruments (PHQ-9, CES-D) for future research to examine specific symptom domains and severity classification.

These revisions transform our original brief limitation statements into a comprehensive discussion that transparently acknowledges methodological constraints while providing concrete directions for future research.

Reviewer #2

Reviewer #2: Good conceptulisation.
1. However, at certain places, refer to the timeline (2020 vs. 2021) instead of referring to it as the initial phase to enhance readability.

**Response:**

Thank you for this valuable suggestion to enhance clarity. We have systematically revised the entire manuscript to replace vague phase terminology with precise year references.

**1. Global Terminology Revision**

**All vague phase terminology has been replaced with year-specific references:**

- "initial phase" → "2020"
- "escalation phase" → "2021"
- "early pandemic" → "2020"
- "pandemic escalation" → "2021"

**2. Key Section Revisions**

**Materials and Methods (Lines 239-280):**

- Original: "...dividing it into two main phases: the initial and escalation phases"
- Revised: *"...dividing it into two periods: 2020 and 2021"*

**Results Section Headings:**

- "Results in the Initial Phase" → "Results in 2020"
- "Results in the Escalation Phase" → "Results in 2021"

**3. Updated Table Titles**

- Table 4: "Results of COVID-19-Related Depressive Symptoms in 2020"
- Table 5: "Results of COVID-19-Related Depressive Symptoms in 2021"

**4. Contextual Phase References**

Where analytical context requires phase references, we use parenthetical notation:

- Example: "During 2020 (initial phase of the pandemic)"
- This maintains clarity while preserving the analytical framework

2. Are you referring to the cumulative odds? May describe precisely

**Response:**

Thank you for requesting clarification on the terminology. Yes, we are referring to cumulative odds in the context of the proportional odds model.

In the ordered logistic regression analysis used in this study:

- We calculate cumulative odds, defined as P(Y ≤ j) / P(Y > j), which represents the ratio of the probability of being at or below a particular category versus being above that category for each threshold j

- The model operates under the proportional odds assumption, meaning that the effect coefficients (β) remain constant across all cumulative logits, while only the threshold parameters (intercepts) vary across categories

We have revised the manuscript to clarify this terminology. Specifically, we have:

1. Explicitly noted that the ordered logistic model is a proportional odds model

2. Added a precise definition of cumulative odds with the mathematical expression P(Y ≤ j) / P(Y > j)

3. Explained the proportional odds assumption underlying the model

These revisions ensure that readers understand both the specific type of odds being modeled (cumulative odds) and the key assumption (proportional odds) that characterizes this analytical approach.

3. Why are you using a single-item 10-point scale instead of other depression inventories?

**Response:**

We thank the reviewer for this important methodological question. We have substantially revised the Methods section and Limitations section to address this concern.

Validation Evidence Added:

We have added a new paragraph citing four validation studies demonstrating the reliability of single-item depression measures:

- Zimmerman et al. (2006): r=0.78 correlation with 18-item CUDOS

- van Rijsbergen et al. (2014): 94% discriminative accuracy (AUC=0.94)

- Mallon et al. (2002): 81% agreement with 14-item HADS

- Khajuria et al. (2021): Successfully used in pandemic surveillance across 41 countries

Justification for Our Choice:

Our single-item approach was appropriate for pandemic surveillance because it enabled rapid assessment, large-scale feasibility (N=10,000), and explicitly measured pandemic-attributed depression—a distinction not directly captured by PHQ-9 or CES-D. Our measure served population-level monitoring objectives rather than clinical diagnosis.

Limitations Acknowledged:

We revised the Limitations section to explicitly acknowledge that "while our single-item scale has demonstrated validity in epidemiological research, it does not capture the multidimensional nature of depression assessed by comprehensive instruments such as PHQ-9 or CES-D. Future studies may benefit from incorporating more detailed depression assessments."

This response integrates with RR_5, addressing similar concerns from Reviewers 1 and 4.

Reviewer #3

Reviewer #3: Reviewer’s comments
Title: Urban density and depression during COVID-19 in Seoul: Age and phase differences with the moderating role of social participation

Title:
The title is clear, specific, and accurately reflects the study’s core variables (urban density, depression, COVID-19, age, phase, and social participation). It effectively signals the focus on Seoul and the moderating role of social participation.

Background and Rationale:
The introduction starts well by establishing the relevance of the topic (COVID-19’s mental health impact) and identifies clear gaps: temporal distinctions of the pandemic, age-specific differences, and the role of social participation. This gives the study a strong rationale.

Objectives:
The objective is stated clearly to analyze the relationship between depression, urban density characteristics, and the moderating effect of social participation by age and pandemic phase. This is well-aligned with the background.

Methods:
The method is briefly stated: data from 25 Seoul districts during 2020 and 2021, analyzed using ordinal logistic regression. This is appropriate, though it could briefly mention the source of the data or sample size for clarity.

Results:
Key findings are logically presented and match the objectives. The abstract clearly states (1) urban density factors are associated with increased depression, (2) social participation buffers these effects, and (3) effects vary by age especially stronger among older adults. This section is well-organized and impactful.

Conclusion/Implications:
The conclusion ties the findings back to their broader significance, highlighting the need to understand the complex interplay of urban density, social participation, and mental health. This is appropriate but could briefly mention how this understanding can inform public health or urban policy.

Strengths:
• Clear structure (background → aim → methods → results → implications)
• Relevant and timely topic
• Well-articulated findings and moderation analysis

Areas for Improvement:
1. Provide a brief mention of the sample size or participant demographics.

**Response:**

We have enhanced the manuscript with detailed information about sample size, demographics, and data sources.

**1. Sample Size Clarification**

- **Original:** " The Seoul Citizen Survey targeted 5,000 individuals aged 15 years and older residing in Seoul..."
- ***Revised:*** *"* *The Seoul Citizen Survey annually targeted 5,000 individuals aged 15 years and older residing in Seoul, yielding a total sample of 10,000 participants across the two study periods (2020: n=5,000; 2021: n=5,000)."*

**2. Demographic Characteristics**

- **Original:** "These characteristics included sex (male, female), age, educational level..."
- ***Revised:*** *"* *Participants were categorized into three age groups for analysis: young adults (19-34 years), middle-aged adults (35-64 years), and older adults (65 years and above), with sample distributions detailed in Table 2. These characteristics included sex (male, female), age, educational level..."*

**3. Table 2 Enhancement**

- **Original:** "Table 2 illustrates the differences in COVID-19-related depressive symptoms according to year and age group."
- ***Revised:*** *Table 2 presents the sample distribution and differences in COVID-19-related depressive symptoms by pandemic phase and age group. In 2020, the sample comprised 1,227 young adults (19-34 years), 2,695 middle-aged adults (35-64 years), and 1,080 older adults (≥65 years). In 2021, the distribution was 1,302 young adults, 2,697 middle-aged adults, and 1,001 older adults.*

2. Clarify whether depression was measured via self-report surveys, clinical data, or another instrument.

**Response:**

Thank you for this important comment. We have now added validation evidence and justification for the single-item measure.

**1. Validation Evidence Added (Line 290)**

We added a paragraph citing empirical studies demonstrating validity of single-item depression measures in epidemiological research:

- **Zimmerman et al. (2006)**: Strong correlation with 18-item CUDOS (r=0.78, p<0.001) and clinician-rated CGI-S (r=0.64, p<0.001) among 562 outpatients
- **van Rijsbergen et al. (2014)**: 94% discriminative accuracy (AUC=0.94) against structured clinical interviews (SCID-I)
- **Mallon et al. (2002)**: 81% agreement with 14-item HADS in large epidemiological study

**2. Pandemic-Specific Justification Added (Line 295)**

We explained why this approach was appropriate for COVID-19 surveillance:

- **Rapid assessment**: Essential for timely public health response during evolving pandemic
- **Large-scale feasibility**: Minimized respondent burden (N=5,000 per wave)
- **Pandemic-specific focus**: Our measure explicitly assesses depressive symptoms "**due to the pandemic**," differentiating pandemic-attributed depression from general depression
- **International precedent**: Khajuria et al. (2021) successfully used single-item assessment among 2,527 healthcare workers across 41 countries during COVID-19

**3. Distinction from PHQ-9/CES-D**

Our measure serves different objectives than clinical instruments:

- **PHQ-9/CES-D**: Clinical diagnosis and comprehensive symptom assessment
- **Our measure**: Population-level surveillance of pandemic-attributed mental health impacts

This design aligns with municipal health monitoring objectives prioritizing rapid, large-scale assessment over detailed clinical evaluation.

**4. Limitations Acknowledged**

We revised the existing limitation statement to acknowledge measurement constraints while referencing the validation evidence:

**Original:** *"Third, the accuracy of measuring COVID-19-related depressive symptoms was limited. In this study, depressive symptoms were measured using a single-item 10-point scale..."*

**Revised:** *"Third, while our single-item 10-point depression scale has demonstrated validity in epidemiological research (as discussed in Methods section), it does not capture the multidimensional nature of depression assessed by comprehensive instruments such as the PHQ-9 or CES-D. Specifically, this measure may not fully reflect the complex dimensions of depressive symptoms caused by the COVID-19 pandemic. Future studies may benefit from incorporating more detailed depression assessments to examine specific symptom domains and enable clinical severity classification."*

3. Include a sentence on potential policy or practical implications to strengthen the conclusion.

**Response:**

We sincerely thank both reviewers and the editor for this valuable suggestion to strengthen the practical applicability of our findings. We have substantially expanded the Conclusion section by adding three comprehensive paragraphs that translate our empirical results into actionable policy recommendations.

The additions address three key domains:

First, we now provide specific recommendations for urban planning and environmental interventions to mitigate density-related mental health risks during pandemics. These include enhancing access to diverse green spaces (plazas, parks, riverside greenways, mountain belts characteristic of Seoul's landscape), implementing public transportation measures to reduce crowding (increased frequency, real-time occupancy monitoring, enhanced sanitation protocols), and adopting "15-minute city" concepts that strategically locate essential infrastructure within residential neighborhoods to minimize population mobility.

Second, we outline phase-adaptive mental health policies that evolve with pandemic progression. We specify that early-phase interventions should focus on reducing acute fears through clear risk communication and immediate psychological support, while later-phase policies should emphasize rebuilding social networks and facilitating community reintegration programs. Throughout all phases, we recommend actively facilitating safe social engagement opportunities through outdoor activities, neighborhood networks, and digital connectivity programs.

Third, we provide age-specific recommendations addressing the heightened vulnerability of older adults demonstrated in our study. These include establishing accessible community centers within walking distance, implementing volunteer visitor programs, creating safe outdoor spaces designed for older adult mobility, and conducting proactive outreach to identify at-risk older adults in high-density neighborhoods [26,54-57].

These policy recommendations are directly grounded in our study's key findings—particularly the moderating role of social participation, the age-specific vulnerability patterns, and the phase-dependent changes in depression—and are supported by relevant literature. We believe these additions substantially enhance the manuscript's relevance for evidence-based policymaking during infectious disease outbreaks.

Overall Evaluation:
This is a well-written and coherent paper that clearly communicates the study’s purpose, methods, and main findings. With minor additions on data details and implications, it would meet high scholarly standards.

Reviewer #4

Reviewer #4: It is easy to understand from the beginning what the authors' objectives were, sounds and it is easy to read and very understandable. First, I analyzed the main points and then introduced some improvements that could give more consistency to the focused themes.

The consistent association between urban density characteristics (population mobility, residential density, and public transportation congestion) and Mental Health (increased depressive symptoms during the COVID-19 pandemic). Social participation is identified as a key factor in mitigating depressive symptoms caused by urban density. Engaging in social activities can buffer the negative mental health impacts of high-density environments, particularly among middle-aged and older adults. This finding emphasizes the protective role of social connectedness during crises.

Also reveals that the effects of urban density and social participation on depressive symptoms vary significantly by age group. Older adults are more sensitive to urban density stressors and benefit the most from social participation, while younger adults experience different dynamics. The need for age-specific mental health interventions during pandemics.

Shows also that depressive symptoms decreased overall during the escalation phase of the pandemic compared to the initial phase, suggesting societal adaptation over time. Explain how mental health evolves during prolonged crises and the importance of resilience-building measures.

The study identifies significant interaction effects between urban density characteristics and social participation, particularly in mitigating depressive symptoms in high-density environments. For example, supportive neighbourhood relationships in densely populated areas can offset stress caused by crowding. The importance of fostering social networks in urban planning and public health strategies. These ideas contribute to a deeper understanding of the complex relationships between urban density, social participation, and mental health during pandemics, offering valuable guidance for policymakers and urban planners.

Although this article is very well written and demonstrates some concerns about practical applications for future research, some methodological and coherence issues must be more specified.

1. The study uses cross-sectional data from 2020 and 2021, which limits its ability to track changes over time for the same subjects. This restricts the ability to establish causal relationships and observe temporal dynamics. The absence of a longitudinal approach prevents the study from capturing the long-term effects of urban density and social participation on mental health as the pandemic evolved. A longitudinal design would have been more appropriate to observe how depressive symptoms evolved throughout the pandemic and to establish causal relationships between urban density, social participation, and mental health. While some results are statistically significant (e.g., odds ratios for urban density and depressive symptoms), their practical significance may be limited. For example, small changes in odds ratios may not translate into meaningful real-world impacts.

**Response:**

We thank the reviewer for this important methodological observation. This limitation is already explicitly acknowledged in our Limitations section. Specifically, we address three key aspects:

1) We acknowledge that the cross-sectional design restricts our ability to establish causal relationships between urban density, social participation, and depressive symptoms.

2) We clarify that while we compared two distinct pandemic phases (2020 vs. 2021), these represent separate cross-sectional samples rather than longitudinal tracking of the same individuals.

3) We recommend that future research should employ longitudinal designs to track the same individuals across multiple pandemic phases, which would strengthen causal inference about the temporal dynamics between urban density, social participation, and mental health changes.

We believe this important limitation is sufficiently addressed in the current manuscript.

2. The Ordered Logistic Regression Model is suitable for analysing ordinal data, it may not fully capture the complexity of interactions between urban density, social participation, and depressive symptoms. Advanced statistical techniques, such as structural equation modelling, could have provided deeper insights into these relationships.

While the study highlights the role of urban density, it does not explore other potential factors influencing mental health, such as economic disparities, healthcare access, or cultural differences, which could provide a more comprehensive understanding.

**Response:**

We sincerely thank the reviewer for these thoughtful methodological suggestions, which highlight important avenues for advancing research in this area.

Statistical approach: We appreciate the suggestion regarding structural equation modeling (SEM). Our ordered logistic regression with interaction terms was specifically chosen to test our primary research question: whether social participation moderates (i.e., buffers) the relationship between urban density and depression. This approach is well-established for moderation analysis in epidemiological research and directly addresses the buffering mechanism demonstrated in our interaction analyses (Table 3). While we recognize that SEM offers valuable capabilities for exploring mediation pathways—such as examining whether urban density influences depression through changes in social participation levels—this represents a complementary but distinct analytical focus from our moderation framework.

We agree this would be an excellent direction for future research and appreciate the reviewer bringing this to our attention.

Additional variables: We are grateful for the suggestion to consider additional contextual factors. Our models include controls for key sociodemographic variables that could confound the relationships of interest, including age, gender, education, household income, and living arrangements (Table 2). The reviewer's point about economic disparities, healthcare access, and cultural factors is well-taken. While these represent important considerations that could enrich understanding of mental health disparities, they extend beyond the specific scope of our study, which focuses on the interplay between urban environmental context (density) and social resources (participation) during the pandemic.

We fully agree that future research integrating these additional dimensions would provide valuable complementary insights into the multifaceted nature of mental health disparities during infectious disease outbreaks.

We hope these clarifications address the reviewer's concerns while acknowledging the value of the suggested directions for future investigation.

3. The study focuses on 25 districts in Seoul, which has unique urban characteristics such as high population density and an extensive public transportation system. This makes it challenging to generalize the findings to smaller cities, rural areas, or urban environments with different characteristics.

**Response:**

We sincerely thank the reviewer for emphasizing this important generalizability concern. We have substantially strengthened our limitations discussion to explicitly address this issue.

Specifically, we have revised the generalizability limitation statement as follows:

Original: "Second, this study focused on 25 districts in Seoul..."

Revised: " Second, this study was conducted in 25 districts of Seoul, reflecting the city's unique urban context characterized by high population density (16,000 persons/km²) and well-developed public transportation systems. Given these distinctive characteristics differ from other cities and regions, caution is needed when generalizing our findings. Future studies should expand their scope to include a variety of cities of different sizes and characteristics to evaluate the generalizability of the results."

This revision now explicitly acknowledges Seoul's unique characteristics—including specific population density metrics and transportation infrastructure—and clearly states the limitation in generalizing to smaller cities, rural areas, or urban environments with different characteristics. We believe this addresses the reviewer's concern by providing concrete context about Seoul's distinctive urban features and their implications for external validity.

4. Data were collected through in-person and online surveys, which may introduce biases due to differences in response rates or the willingness of participants to engage during the pandemic.

**Response:**

We appreciate this concern. We would like to clarify the data collection procedures based on the official survey report. The primary data collection method was in-person household interviews. Online surveys were only offered as alternatives when respondents refused in-person interviews due to COVID-19 concerns or when household members were unavailable (pp. 7, 17). This design prioritized face-to-face interviews with online options serving primarily as COVID-19 accommodations rather than a parallel primary method.

The survey implemented rigorous quality control measures to minimize potential biases, including: (1) random 30% telephone verification of completed surveys, (2) replacement samples from the same neighborhood and housing type, (3) regression-based non-response imputation, and (4) systematic interviewer training and monitoring (pp. 17-18).

While we acknowledge that survey mode mixing can potentially introduce bias, the design and quality control procedures were specifically intended to minimize such effects.

5. The study measures social participation as a binary variable (participation or no participation), which may not capture the depth, frequency, or quality of social interactions, potentially oversimplifying its impact on mental health.

**Response:**

We thank the reviewer for this important observation. This is indeed a limitation of our study, constrained by the available measures in the Seoul Community Health Survey data.

We have added this limitation to our Limitations section as the fourth point: "Social participation was measured as a binary variable (participation vs. no participation), which does not capture the depth, frequency, or quality of social interactions. Future research with more detailed measures of social participation—including frequency, intensity, and types of activities—would provide deeper insights into how different dimensions of social engagement buffer urban density stressors."

This addition acknowledges the measurement constraint while providing a clear direction for future research to examine more nuanced dimensions of social participation.

6. Depressive symptoms were measured using a single-item 10-point scale. While this approach is convenient for large-scale surveys, it may not fully capture the multidimensional nature of depression, potentially oversimplifying the complex aspects of mental health during the pandemic. Using validated, multidimensional depression scales (e.g., PHQ-9 or CES-D) would have provided a more nuanced understanding of mental health impacts.

**Response:**

Thank you for this important comment. We have now added validation evidence and justification for the single-item measure.

**1. Validation Evidence Added (Line 290)**

We added a paragraph citing empirical studies demonstrating validity of single-item depression measures in epidemiological research:

- **Zimmerman et al. (2006)**: Strong correlation with 18-item CUDOS (r=0.78, p<0.001) and clinician-rated CGI-S (r=0.64, p<0.001) among 562 outpatients
- **van Rijsbergen et al. (2014)**: 94% discriminative accuracy (AUC=0.94) against structured clinical interviews (SCID-I)
- **Mallon et al. (2002)**: 81% agreement with 14-item HADS in large epidemiological study

**2. Pandemic-Specific Justification Added (Line 295)**

We explained why this approach was appropriate for COVID-19 surveillance:

- **Rapid assessment**: Essential for timely public health response during evolving pandemic
- **Large-scale feasibility**: Minimized respondent burden (N=5,000 per wave)
- **Pandemic-specific focus**: Our measure explicitly assesses depressive symptoms "**due to the pandemic**," differentiating pandemic-attributed depression from general depression
- **International precedent**: Khajuria et al. (2021) successfully used single-item assessment among 2,527 healthcare workers across 41 countries during COVID-19

**3. Distinction from PHQ-9/CES-D**

Our measure serves different objectives than clinical instruments:

- **PHQ-9/CES-D**: Clinical diagnosis and comprehensive symptom assessment
- **Our measure**: Population-level surveillance of pandemic-attributed mental health impacts

This design aligns with municipal health monitoring objectives prioritizing rapid, large-scale assessment over detailed clinical evaluation.

**4. Limitations Acknowledged**

We revised the existing limitation statement to acknowledge measurement constraints while referencing the validation evidence:

**Original:** *"Third, the accuracy of measuring COVID-19-related depressive symptoms was limited. In this study, depressive symptoms were measured using a single-item 10-point scale..."*

**Revised:** *"Third, while our single-item 10-point depression scale has demonstrated validity in epidemiological research (as discussed in Methods section), it does not capture the multidimensional nature of depression assessed by comprehensive instruments such as the PHQ-9 or CES-D. Specifically, this measure may not fully reflect the complex dimensions of depressive symptoms caused by the COVID-19 pandemic. Future studies may benefit from incorporating more detailed depression assessments to examine specific symptom domains and enable clinical severity classification."*

7. The study excludes the fifth wave (recovery phase) of the pandemic, which accounted for 95% of cumulative cases. This omission limits the understanding of how mental health evolved during the transition to normality.

**Response:**

We thank the reviewer for this valuable observation. The recovery phase (2022 onwards) would indeed provide important additional insights into mental health trajectories as the population adapted to the pandemic. However, the Seoul Survey discontinued collecting pandemic-specific mental health data in 2022, as residents had largely adapted to the endemic situation.

Our study therefore focused on the initial phase (2020) and escalation phase (2021), when pandemic-related distancing measures and urban density impacts were most pronounced. We agree that examining the recovery phase would be valuable if such data become available in future research.

8. The study uses cross-sectional data, which cannot establish causality assumption. However, some results are interpreted as causal relationships (e.g., urban density causing depressive symptoms), which may be misleading.

**Response:**

Thank you for this crucial methodological point. We have systematically revised the entire manuscript to remove causal language and emphasize associations consistent with our cross-sectional design.

**1. Abstract Revisions (Lines 33-40)**

First finding:

- **Original:** "...were consistently associated with increased depression throughout the pandemic"
- **Revised:** *"...were consistently associated with higher levels of depression across both study periods (2020 and 2021)"*

Second finding:

- **Original:** " social participation demonstrated a buffering effect, mitigating the depressive impact of urban density characteristics regardless of the pandemic phase."
- **Revised:** *" Second, social participation showed a significant moderating association with the relationship between urban density characteristics and depression, regardless of the pandemic "*

**2. Results and Discussion Revisions**

Temporal patterns:

- **Original:** " This finding suggests the possibility of adapting to the pandemic over time. "
- **Revised:** *" This finding indicates a temporal decline in COVID-19-related depressive symptoms during the study period. "*

Policy associations:

- **Original:** " ..., such as encouraging people to stay at home, likely led to increased residential density "
- **Revised:** " *..., such as encouraging people to stay at home, were associated with increased residential density "*
- **Original:** " coupled with stay-at-home policies, can amplify pandemic-related depressive symptoms. "
- **Revised:** *" coupled with stay-at-home policies, were associated with higher pandemic-related depressive symptoms "*
- **Original:** "These results suggest that social participation mitigates infection-related anxiety arising from urban density."
- **Revised:** *"These results suggest that social participation was associated with lower infection-related anxiety arising from urban density."*
- **Original:** "Active social interactions appear to alleviate depressive symptoms even in high-density environments."
- **Revised:** *"Active social interactions were associated with lower depressive symptoms even in high-density environments."*

**4. Conclusion Revisions**

- **Original:** "This suggests that dense urban environments can amplify anxiety about infections."
- **Revised:** *"This suggests that dense urban environments were associated with higher anxiety about infections."*
- **Original:** "playing a role in reducing the increase in depressive symptoms caused by high urban density"
- **Revised:** *"playing a role in reducing the increase in depressive symptoms associated with high urban density"*

It seems that incorrect assumptions bring some methodological limitations to this article, oversimplified measurements, inadequate control of confounding variables, and potential biases in data collection and interpretation. These issues highlight the need for more rigorous and comprehensive approaches in future research.
